# Using Text Mining and Data Visualization Approaches for Investigating Mental Illness from the Perspective of Traditional Chinese Medicine

**DOI:** 10.3390/medicina59020196

**Published:** 2023-01-18

**Authors:** Wan-Ling Lin, Yu-Chi Liang, Kuo-Hsuan Chung, Ping-Ho Chen, Yung-Chun Chang

**Affiliations:** 1 Department of Traditional Chinese Medicine, Taipei Medical University Hospital, Taipei 110, Taiwan; 2 Graduate Institute of Data Science, Taipei Medical University, Taipei 110, Taiwan; 3 Department of Psychiatry, Taipei Medical University Hospital, Taipei 110, Taiwan; 4 Clinical Big Data Research Center, Taipei Medical University Hospital, Taipei 110, Taiwan

**Keywords:** traditional Chinese medicine, mental illnesses, text mining, data visualization, ontology construction

## Abstract

*Background and Objectives.* Anxiety and depressive disorders are the most prevalent mental disorders, and due to the COVID-19 pandemic, more people are suffering from anxiety and depressive disorders, and a considerable fraction of COVID-19 survivors have a variety of persistent neuropsychiatric problems after the initial infection. Traditional Chinese Medicine (TCM) offers a different perspective on mental disorders from Western biomedicine. Effective management of mental disorders has become an increasing concern in recent decades due to the high social and economic costs involved. This study attempts to express and ontologize the relationships between different mental disorders and physical organs from the perspective of TCM, so as to bridge the gap between the unique terminology used in TCM and a medical professional. *Materials and Methods.* Natural language processing (NLP) is introduced to quantify the importance of different mental disorder descriptions relative to the five depots and two palaces, stomach and gallbladder, through the classical medical text Huangdi Neijing and construct a mental disorder ontology based on the TCM classic text. *Results.* The results demonstrate that our proposed framework integrates NLP and data visualization, enabling clinicians to gain insights into mental health, in addition to biomedicine. According to the results of the relationship analysis of mental disorders, depots, palaces, and symptoms, the organ/depot most related to mental disorders is the heart, and the two most important emotion factors associated with mental disorders are anger and worry & think. The mental disorders described in TCM are related to more than one organ (depot/palace). *Conclusion.* This study complements recent research delving into co-relations or interactions between mental status and other organs and systems.

## 1. Introduction

The World Health Organization (WHO) has published alarming data regarding mental disorders, in which depression is listed as one of the main causes of disability worldwide [1,2]. Anxiety and depressive disorders are the most prevalent mental disorders in 2019, affecting 1 in 8 people, or 970 million people worldwide. Due to the COVID-19 pandemic, there were significantly more people in 2020 who suffered from anxiety and depressive disorders as compared to previous years. Increasing numbers of studies have shown that a considerable fraction of COVID-19 survivors have a variety of neuropsychiatric problems that continue or even manifest months after the initial infection. With new variants and lineages continuously evolving, the number of people infected by the virus is expected to break records at an unprecedented rate. As such, the number of people suffering from anxiety and depressive disorders and other neuropsychiatric problems are also expected to reach record highs. Management of these mental disorders are of great urgency and importance, as the expected economic and societal costs may be greater than preliminary forecasts.

Preceding research in the early 20th century on mental disorders had focused on interpreting the relations between emotions and the human body, such as analyzing the manifestation of emotions through observable body reactions [3,4,5,6]. However, the rise of psychiatric research can be traced back to the 19th century, in which Western biomedicine started to emphasize the importance of the brain in the proper or improper functioning of mental activity, and neurology and psychiatry were combined into a single science. This was totally different from TCM, which views the body as an intricately woven system that has to be considered holistically and cannot be compartmentalized. However, the terminology used in TCM involves idioms that only trained professionals can understand, and not the modern scientific language that is typically seen, therefore preventing non-TCM trained professionals from understanding or utilizing the wealth of information in the TCM literature. This study seeks to bridge the gap such that non-TCM professionals may also understand basic TCM teachings in mental disorders. TCM considers mental disorders in three aspects, namely recognition, manifestation, and response. This combination is important as TCM views mental disorders as a result of disharmony between the somatic and/or spiritual entities.

Within the framework of TCM, this is understood as an imbalance of Yin and Yang, Vital Energy (“*Qi*”, “*氣*”), and Blood (“*Shie*”, “*血*”). TCM uses pattern (or syndrome) differentiation to diagnose diseases, which considers signs and symptoms of the disease, the environment, and the patient’s profile, allowing personalized medical treatment. Taking depression as an example, TCM believes that depression is caused by an imbalance of different organs and *Qi*. Mental-like disorders have long been mentioned in the TCM literature, such as the *Huangdi Neijing* (Inner Canon of the Yellow Emperor) [7], the earliest Chinese medical classic that was written between the Warring States Period and the Han Dynasty (from 475 BC to AD 220). This ancient Chinese medical classic presents theories regarding mind, body, causations of disease, reasoning behind affiliating mental emotions, observed symptoms, and physical organs. The mental-like symptoms listed in this classic demonstrate that ancient Chinese physicians were aware of mental disorders, their diagnosis, and management. In this research, we investigate the correlation between mental disorders, symptoms, and organs within the *Huangdi Neijing* through text mining and data visual analytics. The observations and results are interpreted from the perspective of TCM and Western medicine.

The remainder of the work is organized as follows. Section 2 describes our proposed approach in detail. Section 3 presents the experiment results and discussions, and we also detail the process of ontology construction. Finally, we conclude our work in Section 4.

## 2. Materials and Methods

### 2.1. Data Source and Collection

The spirit-mind theory in the Huangdi Neijing (HDNJ) is the foundational source of ancient Chinese Medical Psychology. It elaborates the impact of emotions on physical and mental health. The contents of the HDNJ are expressed in archaic Chinese, and the phrasings of mental disorder symptoms lack consistency, which makes it difficult to analyze. In order to successfully achieve the purpose of this work, words having psychological meaning descriptions are identified through manual review and are then classified; in all, 65 mental disorder descriptions are identified. We then further classified them into nine categories according to the seven emotions theory [7], and referred to [8] for the specification of various symptoms. The nine categories are: anger (nu, 怒), happiness (shi, 喜), worry & think (yousz, 憂思), sadness (bai, 悲), fear & fright (jingkung, 驚恐), sleep disorder (bumei, 不寐), drowsiness (shrshuei, 嗜睡), forgetfulness (jianwang, 健忘), and hallucination (huanjiue, 幻覺). The classifications were discussed and verified by three TCM practitioners, each with more than 10 years of clinical experience.

The dataset was collected from the HDNJ according to descriptions of mental disorders’ symptoms based on TCM teachings. Subsequently, the data and the collected mentions of the visceral systems are classified as different mental disorder symptoms and different viscera accordingly. The data assembled contained multiple groups of passages referring to different categorical mental disorder symptoms with reference to the five depots of liver, heart, spleen, lung, and kidney, and two of the six palaces, stomach and gallbladder. Among the six palaces, these two are considered to be connected to emotions, with the remaining four (small intestine, large intestine, urinary bladder, and triple energizer) to not be found or too infrequently mentioned to be considered. In view of these outcomes, the present study’s analysis involved the five depots and two of the palaces, gallbladder and stomach. Next, we analyzed the co-occurrence between descriptions of mental disorders and the nine mental disorder categories mentioned above. After removing duplicate passages, 273 out of 1521 descriptions from the HDNJ were analyzed in this study.

### 2.2. Text Mining for Correlation Analysis

We analyze the text of the HDNJ to construct an explanatory model of mental disorders. For this purpose, we investigate the correlation between mental disorders, symptoms, and organs (depots/palaces) within the book. We first detect the correlation degree between mental disorders and symptoms. HDNJ is initially decomposed into a set of candidate clauses, each of which is likely to mention a relationship between symptoms and mental disorders. To generate candidate clauses, the clinician compiled a list for a symptom lexicon corresponding to the nine mental disorders. A clause is identified as a candidate clause if it contains a word or phrase from the symptom lexicon. Next, based on the generated candidate sentences, we calculate the correlation between the mental disorder and symptoms. To compute this correlation, an advantageous feature selection method, the log likelihood ratio (LLR), is used to differentiate word co-occurrence. With a dataset containing positive instances, LLR implements Equation (1) to estimate the likelihood with the assumption that the occurrence of a symptom in the clauses of mental disorder is not random.

(1) $$-2\log\left[\frac{p{\left(SL\right)}^{N\left(SL\wedge MI\right)}{\left(1-p\left(SL\right)\right)}^{N\left(MI\right)-N\left(SL\wedge MI\right)}p{\left(SL\right)}^{N\left(SL\wedge \neg MI\right)}{\left(1-p\left(SL\right)\right)}^{N\left(\neg MI\right)-N\left(SL\wedge \neg MI\right)}}{p{\left(SL|MI\right)}^{N\left(SL\wedge MI\right)}{\left(1-p\left(SL|MI\right)\right)}^{N\left(MI\right)-N\left(SL\wedge MI\right)}p{\left(SL|\neg MI\right)}^{N\left(SL\wedge \neg MI\right)}{\left(1-p\left(SL|\neg MI\right)\right)}^{N\left(\neg MI\right)-N\left(SL\wedge \neg MI\right)}}\right]$$

where *MI* denotes the set of mental disorder clauses in the dataset; *N*(*MI*) and *N*(¬*MI*) are the numbers of positive and negative mental disorder clauses, respectively; and *N*(*S*˄*MI*) is the number of positive mental illness clauses containing the symptom lexicons *SL*. The probabilities *p*(*SL*), *p*(*SL*|*MI*), and *p*(*SL*|¬*MI*) are estimated using maximum likelihood estimation, and a symptom lexicon with a large LLR value is thought to be closely associated with the mental illness. Lastly, depending on their LLR values, we rank the symptom lexicons and extract the top 10 to consolidate into a list of the mental disorder symptoms.

Furthermore, we investigated the correlation between symptoms and organs. Based on our observation, symptoms and organs frequently co-occur in a sentence, and for this reason, we adopted a sentence as a unit for exploring the correlation between symptoms and organs, instead of a clause. It is worth noting that multiple organs are usually mentioned in a sentence, which means we need to identify the specific organ(s) related to a particular symptom expression. The addressed issue is an illustration of coreference resolution, which is an essential element in natural language understanding required in numerous advanced natural language processing tasks. To correctly identify the organ in a sentence, we utilized the closest-first algorithm to link the closest antecedent to the anaphora [9]. We examined the contexts between an organ, o_i_, and a symptom, s_j_, in a sentence extracted from a candidate clause. Only if there are no other organ mentions occurring between o_i_ and s_j_ are they considered positive instances (i.e., symptom s_j_ belongs to organ o_i_); otherwise, sentences are classified as negative instances. For instance, the sentence “The liver stores the blood, and the blood houses the ethereal soul. Vacuity of liver-vital energy (Qi) begets fear, while repletion begets anger” (肝藏血, 血舍魂, 肝氣虛則恐, 實則怒) is classified as a positive instance for the symptom–organ pair liver–fear (肝-恐). However, in the clause “the five essences can be incorporated: happiness is begot when incorporated in the heart; sadness is begot when incorporated in the lung; worry is begot when incorporated in the liver; dread is begot when incorporated in the spleen; fear is begot when incorporated in the kidney; these are known as the five incorporations, which occur due to the vacuity of the depots” (五精所並: 精氣並於心則喜, 並於肺則悲, 並於肝則憂, 並於脾則畏, 並於腎則恐, 是謂五並, 虛而相併者也), there are two depots (spleen “脾” and kidney “腎”) that are mentioned between the depot of liver (肝) and the symptom of fear (恐). We therefore recognize this sentence to be a negative instance. By examining all symptom–organ pairs in the HDNJ, we obtain a sentence set *S* = {*s*_1_, …, *s_m_*}, which contains 388 sentences that express relationships between symptoms and depots and palaces. After this step, the correlation degree is estimated using the LLR feature selection approach. We can further visualize the relationship between mental disorders, symptoms, and the depots/palaces to better comprehend mental disorders from the perspective of TCM.

## 3. Results

We analyze relationships between mental disorder descriptions and illness categories, as well as between illness and the depots and palaces according to TCM. We utilize normalized LLR values for the frequency of co-occurrences between the nine illness categories and the seven organs, which included the five depots (heart, lung, liver, spleen, and kidney) and two palaces (stomach and gallbladder). Table 1 shows the correlation between the mental disorders and their top 10 descriptions. As shown in Table 2, the diverse descriptions of mental symptoms can be classified into emotions. Using the Heart as an example, the description “upset” corresponds to worry & think; “heart feel sad”, “keeps feeling sad,” and “not happy at first,” “easy to be sad” can correspond to sadness; “laughing and can’t stop laughing” can correspond to happiness; while “fear and be alert to be caught” corresponds to fear & fright. We further categorize descriptions into nine mental disorders, and then calculate the distribution for organs. Appendix A shows the complete list of symptoms and their corresponding mental disorders.

**Figure 1:**
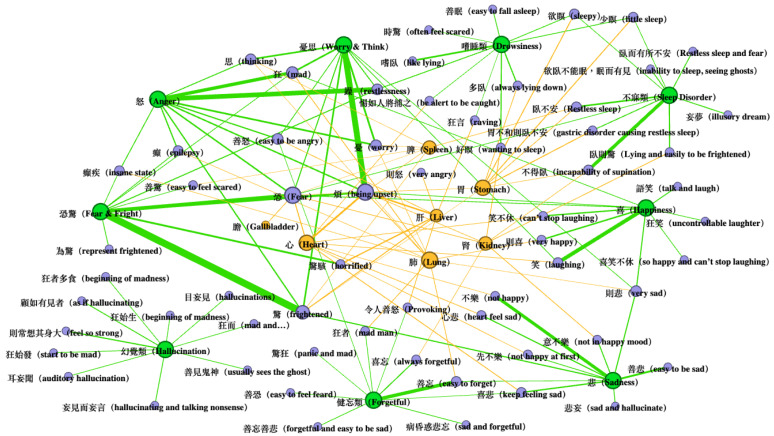
Overview of mental disorder network of Huangdi Neijing.

To better understand the mental disorders mentioned in HDNJ, we constructed an association network to visualize the relationship using Table 1 and Table 2. The node and edge size are determined by the degree of the node and LLR value between two nodes, respectively. Figure 1 presents this network, in which green nodes represent the mental disorders, orange nodes represent the depots/palaces, and purple nodes represent the symptoms. As shown in Figure 1, fear & fright had a high co-occurrence with fright and fear, and these symptoms are highly associated with the liver, kidney, heart, and gallbladder. In addition, we observe that anger is highly associated with the liver and the lung. Worry & think has a high co-occurrence with frustration and worry, and these symptoms are highly associated with the spleen, liver, and lung. Therefore, worry & think is highly associated with the spleen, liver, and lung. This implies that anxiety is related to liver, spleen, heart, and gallbladder, and thus appears to be influenced by multiple organs. Furthermore, depression is often fostered by emotional fluctuations, which are typically related to the liver. Liver vital energy (“ganqi”, “肝氣”) is responsible for the depressive episodes. Mania (“kuang”, “狂”) is found to be associated to the liver and lung. The reversed flow of vital energy is the general cause of mania.

Data visualization of the mental disorder network of Huangdi Neijing suggests that mental disorders are related to multiple organs. Different illnesses have common accompanying symptoms, which can also be seen in the field of psychiatry, where anxiety and depression often have common clinical symptoms.

## 4. Discussion

**Figure 2:**
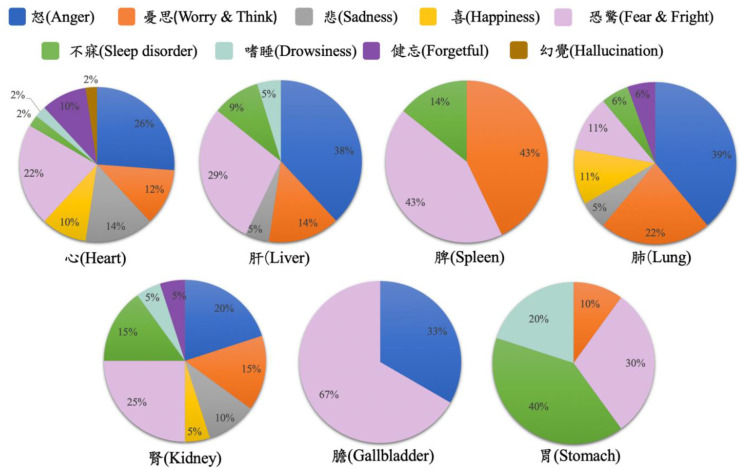
Mental disorder distribution for organs.

**Figure 3:**
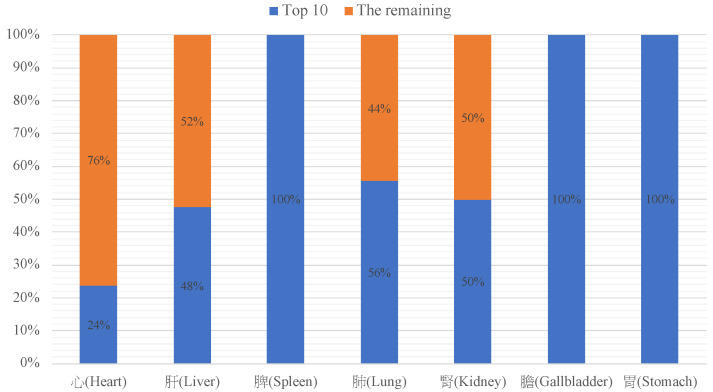
The ratio of top 10 descriptions of the five depots and two palaces.

Figure 2 displays the distribution for organs (depots and palaces). The percentages of various illnesses in relation to the organs are seen. The heart is noticeably prevalent. Anger is highly related to the lung and liver, worry & think is related to the spleen, sadness and forgetfulness are related to the heart, happiness is related to the lung and heart, fear & fright is related to the gallbladder, sleep disorder and drowsiness are related to the stomach, and hallucination may be associated with the heart. Two main factors of mental disorders are anger and worry & think. Figure 3 shows the ratio of the top 10 descriptions of the seven organs. The heart, liver, lung, and kidney have a high ratio of other symptoms not included in the top 10 descriptions. This explains why, in Table 2, the heart and kidney did not have any descriptions about anger in the top 10, while Figure 3 still shows the distribution of anger in these two depots (20%; 26%).

In biomedicine, the heart is an organ that pumps blood throughout the body, providing oxygen and nutrients for bodily functions. Meanwhile, in TCM, the heart is responsible for the regulation of mental activities [10,11,12]. TCM views the heart as being in control of blood circulation, regulating blood vessels, and storing the spirit (“shen”, “神”), which implies consciousness and other cognitive functions [10,11,12,13]. Internal organs rely on the heart and blood for sufficient nourishment. The heart is comprised of heart qi, heart yang, heart blood, and heart yin. A dysfunction in the heart may result in poor circulation and, thus, generates symptoms such as coldness of limbs, and a white or purple complexion [10]. Moreover, previous studies [14,15] have evidenced that heart problems may double the risk of cognitive impairment without memory impairment [16,17]. Previous studies have also suggested the possibility of comorbidity between cardiovascular diseases and mental disorders. In [18,19], the authors demonstrated that chronic mental disorders reduce life span by speeding up exposure to etiological factors that contribute to cardiovascular diseases. The authors of [20,21,22] analyzed the interactions between cardiovascular medication and mental disorders. This study once again reiterates the heart to be the dominant organ related to mental disorders. Western medicine believe that the human brain governs psychological activities; however, TCM holds the view that the heart governs the blood circulation and, therefore, is responsible for the balance of cognitive activities. The influence of the heart on the cognitive activities may be due to the requirement of good blood circulation to maintain the functioning of organs.

It is interesting to note that the liver had a variety of descriptions in fear & fright and anger. In TCM, the liver is in charge of determining the dispersion in all depots and palaces, including the smooth flow of vital energy, blood, and fluid in all directions within the body. Therefore, diseases arise when emotions, especially anger and fear & fright, become too intense or prolonged. These overly intense or prolonged emotions are risk factors of the dysfunction of vital energy, interrupting the balance of yin–yang in the corresponding organs, and eventually bringing about various diseases. In Western medicine, the liver plays an important role in digestion, metabolism, detoxification, and the production of bile. Some studies have implied that, for depression, the liver is presumed to be related to the neuroendocrine system [23,24]. In addition, in Figure 2 the gallbladder seems to have high specificity with fear & fright and anger. The liver and gallbladder seem to be highly associated with these two mental disorders, and future studies may venture into this area.

According to the HDNJ, the spleen and stomach are highly associated with sleep disorder. Recent studies have shown that many gastrointestinal diseases can lead to poor sleep [25,26,27], and cytokines, such as interleukin-1 and interleukin-6 [25,28,29], have been shown to be associated with sleep dysfunction. Interaction between the brain and the gastrointestinal system is of importance in the regulation of the digestive tract and the function of the gut immune system. Animal studies have also found that seemingly insignificant microbiome and unrelated organs, such as the intestines and the brain, can be connected through the gut–brain axis [29,30] and affect the mood of mice, causing anxiety-like or depression-like symptoms [31]. Sleep disorder is closely related to mental health. Many patients with mental disorders also have sleep disorder. In TCM, sleep disorder is closely related to the function of the spleen and stomach. The authors of [7] indicate that “stomach disharmony leading to restless sleep” is an important reason behind sleep disorder. This new thinking may be of benefit to gastroenterologists as addressing the relationship between sleep disorder and gastrointestinal diseases may lead to better patient care.

In Ots’s research [32], out of 243 cases at a TCM clinic, 106 patients were identified as suffering from some type of mental disorder, with approximately two-thirds of all mental health issues directly or indirectly influenced by the liver or the heart, indicating that emotional changes and organ malfunctions may influence each other. Furthermore, in TCM, when a patient is suffering from psychological issues (e.g., depression), they usually manifest as physiological dysfunction of the related organ (e.g., chest tightness with obstructed breathing due to excessive worrying, which therefore hurts the lung). TCM identifies the imbalance of the internal organs by observing changes in the external manifestation. As shown in Figure 2, mental disorders are related to multiple organs rather than a single one (e.g., anger can impact the lung (39%), the liver (38%), the gallbladder (33%), the heart (26%), and the kidney (20%). This encourages clinicians to consider the mutual influence of various organs during management of mental disorders.

## 5. Conclusions

This study provides insight into the TCM’s mental disorder ontology, which presents a different perspective to Western medicine, allowing non-trained professionals to understand the terminology used in TCM. Psychologizing and somatization are often culture-bound narratives. Further study into the psychopathology models of TCM may help broaden the definition of normalcy in Western biomedicine, which can in turn facilitate greater communication and collaboration. This can ultimately improve public health and provide better care to patients. A comprehensive understanding of the integration of the features and diagnostic approaches of Western and TCM remains incomplete. This may improve the clinical management of different mental disorder factors through application of both TCM and Western concepts. The integration and implementation of TCM and Western approaches for specific types of mental disorders can be a fruitful direction.

The present study has various restrictions. Firstly, the descriptions of mental disorders are from a single literature source with finite bibliographical data. It is necessary to broaden the dataset using other classical texts. Secondly, the current data only allow for learning of the relationship between mental disorder descriptions and the depots/palaces. Research needs to be carried out to focus on the associations between cognitive activities and body variations, together with physical symptoms and vital energy movements. In conclusion, extensive studies are needed to promote the integration of TCM and other contemporary concepts to improve the management and treatment of psychosocial disorders and mental disorders in the future.

## Figures and Tables

**Table 1:** Characteristics of the relationships between mental disorder categories and the top 10 mental disorder descriptions.

Anger (怒)	Worry & Think (憂思)	Happiness (喜)
躁_restlessness_ (47.73) 狂_mad_ (33.58) 憂_worry_ (15.32) 恐_fear_ (12.49) 煩_being upset_ (12) 驚_frightened_ (10.64) 善怒_easy to be angry_ (10.02) 癲_epilepsy_ (9.75) 思_thinking_ (7.14) 癲疾_insane state_ (6.39)	煩_upset_ (61.65) 憂_worry_ (16.29) 狂_mad_ (15.01) 思_thinking_ (14.71) 驚_frightened_ (14.46) 恐_fear_ (8.09) 躁_restlessness_ (7.2) 善怒_easy to be angry_ (5.28) 笑_laughing_ (4.79) 則悲_very sad_ (3.72)	笑_laughing_ (38.29) 則喜_very happy_ (12.33) 笑不休_cannot stop laughing_ (10.86) 則悲_very sad_ (8.16) 語笑_talk and laugh_ (6.89) 恐_fear_ (4.93) 狂_mad_ (4.19) 狂笑_uncontrollable laughter_ (4.18) 喜笑不休_so happy and cannot stop laughing_ (4.18) 煩_being upset_ (4.17)
Sadness (悲)	Drowsiness (嗜睡)	Sleep Disorder (不寐)
不樂_not happy_ (31.15) 善悲_easy to be sad_ (17.05) 喜悲_keep feeling sad_ (10.64) 驚_frightened_ (9.17) 心悲_heart feels sad_ (9.16) 則悲_very sad_ (8.61) 意不樂_not in a happy mood_ (7.64) 悲妄_sad and hallucinate_ (5.63) 先不樂_not happy at first_ (4.96) 善忘_easy to forget_ (3.17)	嗜臥_like lying_ (15.19) 多臥_always lying down_ (9.48) 好瞑_wanting to sleep_ (8.6) 躁_restlessness_ (6.57) 善眠_easy to fall asleep_ (5.18) 少瞑_less sleep_ (5.18) 欲瞑_sleepy_ (5.18) 惕如人將捕之_be alert to be caught_ (3.06) 時驚_often feel scared_ (2.69) 狂言_raving_ (1.74)	不得臥_incapability of supination_ (33.45) 臥不安_restless sleep_ (13.61) 臥則驚_lying and easily to be frightened_ (9.95) 臥而有所不安_restless sleep and fear_ (6.6) 妄夢_illusory dream_ (6.6) 胃不和則臥不安_gastric disorder causing restless sleep_ (6.6) 欲臥不能眠,眠而有見_inability to sleep, seeing ghosts_ (6.6) 少瞑_less sleep_ (3.9) 欲瞑_sleepy_ (3.9) 恐_fear_ (3.72)
Fear & Fright (恐驚)	Forgetful (健忘)	Hallucination (幻覺)
驚_frightened_ (74.1) 恐_fear_ (37.61) 煩_upset_ (19.66) 驚駭_horrified_ (16.99) 躁_restlessness_ (7.72) 為驚_represent frightened_ (5.48) 癲_epilepsy_ (4.98) 狂_mad_ (4.28) 癲疾_insane state_ (4.06) 善驚_easy to feel scared_ (3.57)	善忘_easy to forget_ (34.1) 喜忘_always forgetful_ (7.83) 善忘善悲_forgetful and easy to be sad_ (4.52) 病昏惑悲忘_sad and forgetful_ (4.52) 狂者_mad man_ (3.77) 善恐_easy to feel scared_ (3.08) 驚狂_panic and mad_ (2.9) 善怒_easy to be angry_ (2.54) 令人善怒_provoking_ (2.45) 喜悲_easy to be sad_ (2.45)	則常想其身大_feel so strong_ (9.45) 顧如有見者_as if hallucinating_ (6.69) 妄見而妄言_hallucinating and talking nonsense_ (5.66) 狂始生_beginning of madness_ (4.12) 耳妄聞_auditory hallucination_ (4.12) 狂者多食_madness and eating a lot_ (4.12) 善見鬼神_usually sees the ghost_ (4.12) 狂始發_start to be mad_ (4.12) 目妄見_hallucinations_ (4.12) 狂而_mad and…_(3.8)

**Table 2:** Characteristics of the relationships between the five depots and two palaces and top 10 mental disorders descriptions.

Heart (心)	Liver (肝)	Spleen (脾)
煩_being upset_ (15.74) 恐_fear_ (6.93) 心悲_heart feel sad_ (4.03) 笑_laughing_ (2.75) 喜悲_keep feeling sad_ (2.67) 笑不休_cannot stop laughing_ (2.67) 先不樂_not happy at first_ (2.67) 惕如人將捕之_be alert to be caught_ (2.67) 善悲_easy to be sad_ (2.67) 不樂_not happy_ (1.79)	煩_being upset_ (12.42) 驚駭_horrified_ (9.81) 驚_frightened_ (6.56) 其動為語_become talkative_ (4.78) 狂言_raving_ (4.13) 惡言_evil word_ (4.13) 則怒_very angry_ (4.13) 令人善怒_provoking_ (4.13) 欲驚_take fright_ (4.13) 善怒_easy to be angry_ (3.31)	臥不安_restless sleep_ (2.51) 煩_being upset_ (1.66) 驚_frightened_ (1.47) 思_thinking_ (1.45) 善驚_easy to feel feared_ (0.51) 憂_worry_ (0.48) 恐_fear_ (0.02)
Lung (肺)	Kidney (腎)	Gallbladder (膽)
狂_mad_ (4.77) 狂者_mad man_ (4.3) 煩_being upset_ (4.24) 善忘_easy to forget_ (3.6) 則喜_so happy_ (2.65) 癲_epilepsy_ (1.98) 癲疾_insane state_ (1.98) 憂_worry_ (1.36) 思_thinking_ (1.06) 則悲_very sad_ (0.97)	好瞑_like to sleep_ (4.57) 意不樂_not in a happy mood_ (4.57) 臥則驚_lying and easily to be frightened_ (4.09) 恐_fear_ (3.58) 煩_being upset_ (2.85) 不得臥_inability of supination_ (2.4) 惋_sigh_ (2.01) 喜忘_always forgetful_ (2.01) 夜臥則驚_lying at night and easily to be frightened_ (2.01) 病善言_chattering_ (2.01)	病善言_chattering_ (6.04) 為恐_represent fear_ (5.01) 恐_fear_ (3.65)
Stomach (胃)		
少瞑_little sleep_ (6.58) 欲瞑_sleepy_ (6.58) 胃不和則臥不安_gastric disorder causing restless sleep_ (6.58) 多臥_always lying down_ (3.88) 臥不安_restless sleep_ (3.88) 為恐_represent fear_ (2.91) 不得臥_inability of supination_ (1.54)		
恐_fear_ (0.15) 驚_frightened_ (0.07) 煩_being upset_ (0.07)		

## Data Availability

Not applicable.

## Appendix A

**Table A1:** List of symptoms and their corresponding mental disorders.

Symptom	**Mental Disorders**
狂 (madness)	怒 (Anger), 憂思 (Worry & Think), 喜 (Happiness), 恐驚 (Fear & Fright)
憂 (worried)	怒 (Anger), 憂思 (Worry & Think), 喜 (Happiness), 恐驚 (Fear & Fright)
恐 (fear)	怒 (Anger), 悲 (Sadness), 喜 (Happiness), 恐驚 (Fear & Fright), 不寐 (Sleep Disorder)
善怒 (easy to be angry)	怒 (Anger), 恐驚 (Fear & Fright)
譫妄 (delirium)	怒 (Anger), 嗜睡 (Drowsiness)
思 (thinking)	怒 (Anger), 憂思 (Worry & Think)
癲疾 (insane state)	怒 (Anger), 憂思 (Worry & Think), 恐驚 (Fear & Fright)
驚 (frightened)	怒 (Anger), 憂思 (Worry & Think), 悲 (Sadness), 恐驚 (Fear & Fright)
其動為語 (become talkative)	怒 (Anger)
癲疾厥狂 (insane state and syncope)	怒 (Anger)
憂思 (worried thoughtfulness)	憂思 (Worry & Think)
惋 (sigh)	憂思 (Worry & Think)
惋則惡人 (sigh and don’t like seeing people)	憂思 (Worry & Think)
善恐 (easy to feel feared)	憂思 (Worry & Think), 健忘 (Forgetful)
為恐 (represent fear)	憂思 (Worry & Think)
不樂 (not happy)	悲 (Sadness)
善悲 (easy to be sad)	悲 (Sadness), 恐驚 (Fear & Fright)
喜悲 (keep feeling sad)	悲 (Sadness)
意不樂 (not in a happy mood)	悲 (Sadness)
心悲 (heart feel sad)	悲 (Sadness)
先不樂 (not happy at first)	悲 (Sadness)
悲妄 (sad and have illusion)	悲 (Sadness)
則悲 (so sad)	悲 (Sadness), 喜 (Happiness)
笑 (laughing)	喜 (Happiness)
則喜 (so happy)	喜 (Happiness)
喜笑不休 (so happy and cannot stop laughing)	喜 (Happiness)
語笑 (talk and laugh)	喜 (Happiness)
笑不休 (cannot stop laughing)	喜 (Happiness)
狂笑 (guffaw)	喜 (Happiness)
驚駭 (horrified)	恐驚 (Fear & Fright)
善驚 (easily feared)	恐驚 (Fear & Fright)
不得臥 (incapability of supination)	恐驚 (Fear & Fright), 不寐 (Sleep Disorder)
臥不安 (restless sleep)	不寐 (Sleep Disorder)
臥則驚 (lying and easily to be frightened)	不寐 (Sleep Disorder)
臥而有所不安 (restless sleep and fear)	不寐 (Sleep Disorder)
欲臥不得眠, 眠而有見 (can’t sleep and seeing phantom)	不寐 (Sleep Disorder)
胃不和則臥不安 (gastric disorder causing restless sleep)	不寐 (Sleep Disorder)
妄夢 (illusory dream)	不寐 (Sleep Disorder)
夜臥則驚 (lying at night and easily to be frightened)	不寐 (Sleep Disorder)
少瞑 (less sleep)	不寐 (Sleep Disorder), 嗜睡 (Drowsiness)
嗜臥 (like lying)	嗜睡 (Drowsiness)
好瞑 (like to sleep)	嗜睡 (Drowsiness)
多臥 (always lie down)	嗜睡 (Drowsiness)
善眠 (easy to fall asleep)	嗜睡 (Drowsiness)
惕如人將補之 (be alert to be caught)	嗜睡 (Drowsiness)
時驚 (often feel scared)	嗜睡 (Drowsiness)
狂言 (raving)	嗜睡 (Drowsiness)
欲瞑 (sleepy)	嗜睡 (Drowsiness)
善忘 (easy to forget)	健忘 (Forgetful)
喜忘 (always forgetful)	健忘 (Forgetful)
善忘善悲 (forgetful and easy to be sad)	健忘 (Forgetful)
病昏或悲忘 (sad and forgetful)	健忘 (Forgetful)
喜悲 (keep feeling sad)	健忘 (Forgetful)
令人善怒 (provoking)	健忘 (Forgetful)
心悲 (heart feel sad)	健忘 (Forgetful)
狂而 (mad and…)	健忘 (Forgetful), 幻覺 (Hallucination)
目妄見 (see the illusion)	健忘 (Forgetful), 幻覺 (Hallucination)
則常想其身大 (feel so strong)	幻覺 (Hallucination)
顧如有所見 (as if see the illusion)	幻覺 (Hallucination)
妄見而妄言 (see the illusion and talk nonsense)	幻覺 (Hallucination)
善見鬼神 (usually see the ghost)	幻覺 (Hallucination)
狂始發 (start to be mad)	幻覺 (Hallucination)
耳妄聞 (hear the auditory hallucination)	幻覺 (Hallucination)
狂者多食 (mad and eat a lot)	幻覺 (Hallucination)
狂使生 (at beginning of mad)	幻覺 (Hallucination)
